# The Neurobiological Basis of Cognitive Side Effects of Electroconvulsive Therapy: A Systematic Review

**DOI:** 10.3390/brainsci11101273

**Published:** 2021-09-26

**Authors:** Adriana Bassa, Teresa Sagués, Daniel Porta-Casteràs, Pilar Serra, Erika Martínez-Amorós, Diego J. Palao, Marta Cano, Narcís Cardoner

**Affiliations:** 1 Mental Health Department, Unitat de Neurociència Traslacional, Parc Tauli University Hospital, Institut d’Investigació i Innovació Sanitària Parc Taulí (I3PT), 08208 Sabadell, Spain; abassa@tauli.cat (A.B.); tsagues@tauli.cat (T.S.); dporta@tauli.cat (D.P.-C.); pserram@tauli.cat (P.S.); emartineza@tauli.cat (E.M.-A.); dpalao@tauli.cat (D.J.P.); 2 Department of Psychiatry and Forensic Medicine, Universitat Autònoma de Barcelona, 08193 Bellaterra, Spain; 3 CIBERSAM, Carlos III Health Institute, 28029 Madrid, Spain; 4 Department of Psychobiology and Methodology of Health Sciences, Universitat Autònoma de Barcelona, 08193 Bellaterra, Spain

**Keywords:** electroconvulsive therapy, ECT, memory, cognitive impairment, side effects, MDD, biomarkers, hippocampus, NSE, S-100

## Abstract

Decades of research have consistently demonstrated the efficacy of electroconvulsive therapy (ECT) for the treatment of major depressive disorder (MDD), but its clinical use remains somewhat restricted because of its cognitive side effects. The aim of this systematic review is to comprehensively summarize current evidence assessing potential biomarkers of ECT-related cognitive side effects. Based on our systematic search of human studies indexed in PubMed, Scopus, and Web of Knowledge, a total of 29 studies evaluating patients with MDD undergoing ECT were reviewed. Molecular biomarkers studies did not consistently identify concentration changes in plasma S-100 protein, neuron-specific enolase (NSE), or Aβ peptides significantly associated with cognitive performance after ECT. Importantly, these findings suggest that ECT-related cognitive side effects cannot be explained by mechanisms of neural cell damage. Notwithstanding, S-100b protein and Aβ40 peptide concentrations, as well as brain-derived neurotrophic factor (BDNF) polymorphisms, have been suggested as potential predictive biomarkers of cognitive dysfunction after ECT. In addition, recent advances in brain imaging have allowed us to identify ECT-induced volumetric and functional changes in several brain structures closely related to memory performance such as the hippocampus. We provide a preliminary framework to further evaluate neurobiological cognitive vulnerability profiles of patients with MDD treated with ECT.

## 1. Introduction

Electroconvulsive therapy (ECT) is the most effective treatment for major depressive disorder (MDD) [1]. However, concerns about ECT-related cognitive side effects (i.e., disorientation, anterograde amnesia, and retrograde amnesia) are still limiting its prescription to a reduced subgroup of patients with several therapeutic failures [2]. Although different treatment optimization strategies (such as unilateral electrode placement, shorter pulse width, or longer periods between sessions) have been implemented as an attempt to reduce these ECT-related cognitive side effects, the large interindividual neurobiological variability among patients treated with ECT is still leading to inconsistent findings [3,4]. In this context, understanding the neurobiological underpinnings of ECT-related cognitive side effects is a relevant critical step towards the identification of potential predictive biomarkers capable of characterizing those patients who are more susceptible to experiencing these cognitive side effects.

Recent research advocated that the mechanism of action of ECT may be explained by neuroplastic changes such as neurogenesis, synaptogenesis, dendritic sprouting, and glial cell proliferation [5]. In this sense, previous preclinical studies using high-performance liquid chromatography (HPLC) have revealed that repetitive electroconvulsive seizures (ECS) induce restorative changes in hippocampal glutamate/gamma-aminobutyric acid (Glu/GABA) imbalance and N-methyl-D-aspartate (NMDA) receptors [6,7], as well as an up-regulation of the brain-derived neurotrophic factor precursor (proBDNF) and the mature BDNF ratio [8]. Nevertheless, these neuroplastic mechanisms have consistently shown a positive effect on memory and executive functions [9], while ECT has been typically associated with the above-mentioned cognitive side effects. Therefore, a second mechanism of action appears to be simultaneously manifesting during the implementation of ECT. Indeed, other preclinical reports have postulated that ECS results in short-term cognitive alterations through a variety of neurobiological mechanisms such as transient disruption of the blood–brain barrier [10], fluid cell switching, and altered cerebral blood flow [5].

In recent decades, significant advances have been made in the field of acquisition and analysis of structural and functional data of the human brain. Nowadays, there are different sources able to provide data concerning how the human brain works (for extended information, see [11]). In brief, from the registration of the brain electrical activity using electroencephalography (EEG) to the measure of structural and functional brain data by magnetic resonance imaging (MRI) techniques based on the magnetization properties of atomic nuclei, these advances have made it possible to identify not only the key brain structures and circuits altered in mental disorders but also the restorative changes that occur due to the application of different therapeutic strategies such as ECT.

Notwithstanding, since a direct extrapolation of these preclinical findings to real clinical settings is far from being established, a comprehensive review of the neurobiological correlates of ECT-related cognitive side effects in humans is needed to be able to include this knowledge in everyday clinical practice. Therefore, our main objective is to systematically review the current literature exploring potential biomarkers associated with cognitive dysfunction of patients suffering from a depressive episode treated with ECT.

## 2. Materials and Methods

A systematic search of human studies was conducted on PubMed Online (Ovid Technologies, New York, NY, USA), Scopus (Elsevier, Amsterdam, The Netherlands), and Web of Knowledge (Thomson Scientific Technical Support, New York, NY, USA) using the following terms: “ECT cogniti*” plus “impairment” or “disturbances” or “deficits”. Data were collected in accordance with the Preferred Reporting Initiative for Systematic Reviews and Meta-Analyses (PRISMA) [12]. Specifically, the search was performed by two of the authors (A.B. and T.S.), and after reviewing titles, abstracts, and full articles, they resolved disagreements via discussion to reach a consensus. The review protocol was registered in the Prospective International Registry of Systematic Reviews (PROSPERO, CRD4202).

Eligibility criteria.

Articles written in a language other than English, research based on non-human samples (i.e., methodological and laboratory-based studies using cell lines or other organisms), and case reports and studies evaluating patients with a diagnosis other than uni- or bipolar depression or that were irrelevant to the research question (i.e., did not evaluate the neurobiological underpinnings of ECT-related cognitive side effects) were excluded. In addition, we only included studies in which the association between neurobiological markers and cognitive dysfunction was specifically assessed using standardized scales. No exclusion criteria based on sex, age, or date were set.

**Figure 1:**
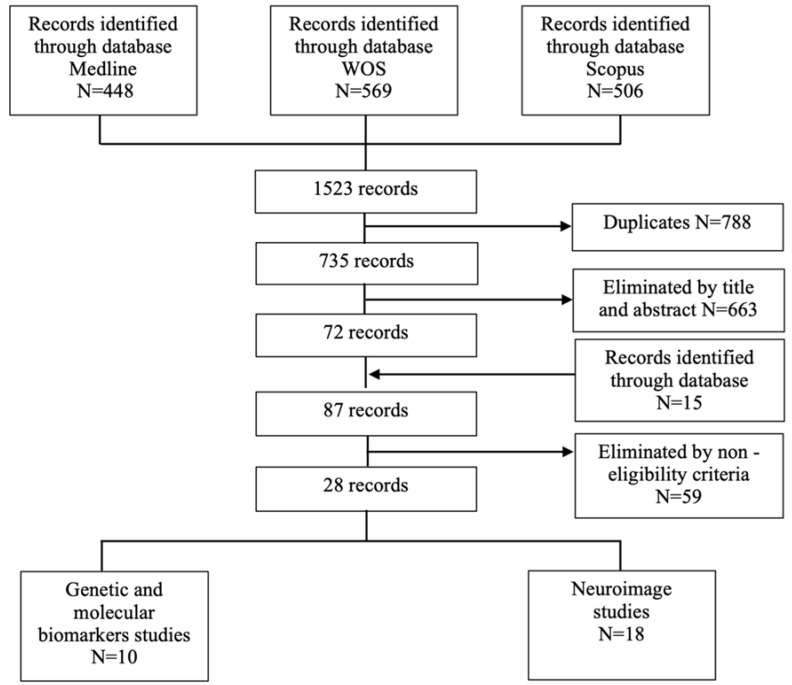
PRISMA block diagram depicting study search and selection process. WOS, Web of Knowledge.

As displayed in Figure 1, a total of 28 articles were included in our systematic review. Importantly, an initial literature evaluation allowed us to identify two main biomarkers categories: (1) genetic and molecular biomarkers and (2) neuroimaging-based biomarkers.

In addition, we used a modified quality index for each article reviewed created for a previous review conducted by research team members [13] that it is based on an adaptation of the Scottish Intercollegiate Guidelines Network [14] as well as multiple imaging-specific assessment criteria used in previous meta-analytic research [15]. For more detailed information please see Table S1 at Supplementary Materials.

## 3. Results

### 3.1. Genetic and Molecular Biomarkers

These results are comprehensively shown in Table 1.

#### 3.1.1. Protein S-100 and Neuron-Specific Enolase (NSE)

Agelink et al. [16] extracted blood samples to evaluate NSE and S-100 concentrations changes in depressed and schizoaffective depressive patients who received ECT. They did not find a significant average increase in either NSE or S-100 concentrations at any time during the course of an ECT series. However, the authors described that those patients with higher post-ECT S-100 values exhibited better cognitive performance. In agreement with these results, Palmio et al. [17] did not observe significant changes in NSE levels after ECT. However, they found a trend towards a significant increase in S-100 beta (S-100b) levels at 2 h after ECT. Notwithstanding, the S-100b concentration increase did not correlate with the Mini-Mental State Examination (MMSE) scores. Likewise, Kranaster et al. [18] also assessed cognitive impairment by means of MMSE evaluations in a sample of patients suffering from MDD. Although they did not find a significant change in NSE levels after ECT, the NSE concentration at baseline was negatively correlated with the MMSE score at baseline. In contrast, Arts et al. [19] reported a significant increase in S100-beta (S100-b) levels 1 h after ECT, with a similar but reduced effect size at 3 h after ECT. In addition, they found that a higher S100-b concentration at baseline was associated with poorer memory function and less subjective cognitive impairment at 5 and 30 days of follow-up. Nevertheless, they failed to demonstrate the relationship between changes in S100-b values during ECT and cognitive performance after ECT.

#### 3.1.2. Aβ Peptides

Piccini et al. [20] found that the plasma Aβ40 and Aβ42 levels, as well as the Aβ40/Aβ42 ratio, were similar at baseline and one week after the last session of ECT. However, correlation analyses showed that the plasma Aβ40 levels were negatively associated with the MMSE total scores at both time points and that the Aβ40/Aβ42 ratio was negatively correlated with the MMSE total score after ECT. In contrast, Kranaster et al. [21] reported an increase in Aβ42 levels in cerebrospinal fluid (CSF) after ECT in those patients who responded to ECT. Nevertheless, the authors did not observe a significant correlation between the Aβ42 concentrations and MMSE scores. In addition, Yamazaki et al. [22] compared two groups of patients with Late-Life Depression (LLD), one treated with pharmacotherapy and the other with pharmacotherapy plus ECT. Although they did not find significant between-group differences in the levels of plasma Aβ40 and Aβ42 at baseline, they detected a marginally significant lower plasma Aβ40 concentration in the pharmacotherapy plus ECT group compared with the pharmacotherapy alone group after ECT. Moreover, their findings also revealed that the plasma Aβ40 levels measured before discharge negatively correlated with the verbal phonetic fluency test (VFT) scores. Interestingly, the authors also performed analyses splitting the sample into three groups: patients with preserved or normal cognition, patients with mild cognitive impairment (MCI) who returned to normal status after treatment (MCI-reverters), and patients with MCI who remained cognitively impaired even after treatment (MCI-non-reverters). They found significantly higher plasma Aβ40 levels at admission in MCI-non-reverters compared with patients cognitively preserved or MCI-reverters. However, no significant between-group differences were observed for plasma Aβ42 levels or Aβ40/A β42 ratio.

#### 3.1.3. Genetics

In the field of genetics, we identified only two articles evaluating potential genetic biomarkers for ECT-related cognitive side effects. Bousman et al. [23] explored COMT Val158Met, DRD2 C957T, BDNF Val66Met, and APOE polymorphisms. They reported a significant effect between DRD2 and BDNF polymorphisms on anterograde memory. In addition, post hoc analyses showed that, among carriers of the BDNF Val/Val genotype, those with the DRD2 TT genotype had significantly less decline in anterograde memory performance than those carrying the TC or CC genotype. Moreover, Ryan et al. [24] hypothesized that shorter telomere length (TL) would predict cognitive side effects after ECT. However, they did not find significant correlations between cognitive performance measures (i.e., global cognition, re-orientation time, and autobiographical memory) and TL.

#### 3.1.4. Cortisol

Neylan et al. [25] hypothesized that higher cortisol levels would predict increased cognitive impairment in depressed patients after receiving ECT. Interestingly, they found that elevated cortisol levels predicted a greater degree of ECT-induced cognitive impairment.

### 3.2. Neuroimaging-Based Biomarkers

#### 3.2.1. Structural Magnetic Resonance Imaging

The results are comprehensively shown in Table 2.

Figiel et al. [26] acquired magnetic resonance imaging (MRI) and computed tomography scans on six patients who developed delirium after ECT. They reported that the whole sample showed white matter hyperintensities (WMH) within the basal ganglia. Notwithstanding, the cross-sectional imaging design of the study did not allow discarding the possibility that these alterations were already shown at baseline. In contrast, Oudega et al. [27] did not detect significant correlations between cognitive functioning and medial temporal lobe atrophy (MTLA). However, a positive trend towards significance was found between cognitive performance and global cortical atrophy (GCA). In this line of work, Wagenmakers et al. [28] evaluated overall brain morphology of patients with LLD before a course of ECT using visual rating scales. They reported that those patients with white matter alterations showed worse MMSE performance at baseline compared to the patients without structural brain abnormalities. Nevertheless, they observed a similar cognitive outcome after ECT regardless of the presence of structural brain alterations before ECT. They also did not find significant correlations between MMSE scores and MTLA or GCA at baseline. Other authors explored, based on previous preclinical studies, the hypothesis that ECT-related cognitive impairment may be related to edema secondary to changes in the blood–brain barrier permeability. Diehl et al. [10] detected a trend towards, a significant correlation between post-ECT medial temporal lobe (MTL) T2 relaxation time increases, and worsened nonverbal anterograde memory scores. Moreover, a post hoc analysis also revealed a significant relationship between left thalamus T2 relaxation time increases and post-ECT verbal and anterograde memory impairment. However, Kunigiri et al. [29] did not find any significant change in T2 relaxation times after ECT.

Cortical thickness was also assessed in two different groups. Gbyl et al. [30] reported cortical thickness increases in 26 brain regions (frontal, temporal and limbic), as well as hippocampal volume increases, six days after ECT. Interestingly, they also observed that these increases returned to baseline measures at six months of follow-up. However, these thickness and volume changes did not correlate with variations in cognitive measures changes. In addition, Xu et al. [31] investigated cortical thickness, surface area, and local gyrification index in a sample of depressed patients before and after ECT. They found that cortical thickness increases in the left inferior parietal gyrus were positively correlated with changes in cognitive scores. Moreover, four additional studies focused their research on the hippocampal volume. Lekwauwa et al. [32] evaluated the relationship between hippocampal volume and cognitive outcome after ECT in a sample of unipolar depressed patients. They found a significant correlation between hippocampal volume decrease and MMSE changes in those patients with moderate/severe cognitive outcomes. In addition, they observed that the left hippocampal volume decreases were above the mean value in participants with moderate or severe memory dysfunction. In contrast, Nordanskog et al. [33] reported a significant increase in hippocampal volume one week after ECT, with values returning to baseline after 6 to 12 months after ECT. Interestingly, the left hippocampal volume increase one week after ECT was positively correlated to Trail Making Test Part A (TMT-A) score, yet this result disappeared after adjusting for the number of ECT sessions. However, Van Oostrom et al. [34] found a significant correlation between the decline of cognitive performance and the hippocampal volume increase. Likewise, Gbyl 2020 et al. [35] analyses showed a significant correlation between an increase in the right dentate gyrus (DG) volume and a decrease in delayed and immediate verbal memory performance, and a significant correlation between an increase in the left DG volume and a decrease in delayed verbal memory performance during ECT. After a 6-month follow-up screening, they found that the decrease in the right DG volume was significantly correlated with the improvement in the delayed memory performance, but they did not detect correlations between cognitive function and the left DG volume. Moreover, they also observed that a decrease in delayed verbal memory performance significantly correlated with the increase in volume in 14 out of 20 hippocampal subregions during ECT and after follow-up and that a decrease in 16 out 20 hippocampal subregions volumes significantly correlated with the improvement in cognitive performance.

#### 3.2.2. Functional Magnetic Resonance Imaging

The results are comprehensively shown in Table 3.

Abbot et al. [36] examined changes in hippocampal resting-state functional connectivity among a sample of unipolar depressed patients before and after treatment with ECT. Specifically, the authors found an increased right hippocampal functional connectivity among ECT responders, which was not statistically significant when compared with healthy controls. They also confirmed, like many others before them, the presence of an aberrant bilateral hippocampal connectivity in depressed patients before the ECT series, including the left MTL and the anterior cingulate cortex (ACC). This abnormal connectivity was no longer evident in comparison with healthy controls after ECT treatment, thus providing confirmatory evidence of normalization. However, the relationship between changes in connectivity and cognition, as measured by differences in percent retention, was not significant. In addition, Bai et al. [37] showed a significant decrease in verbal fluency, which showed a positive association with a decreased functional connectivity between the hippocampus and the bilateral angular gyrus after ECT.

Wang et al. [38] observed a significant decrease in delayed Auditory Verbal Learning Test (AVLT) score in MDD patients after ECT. Interestingly, the impaired memory was closely associated with changes in functional and effective connectivity within the salience network. Similarly, Wang et al. [39] showed a significant reduction in delayed recall Rey AVLT (RAVLT) scores following ECT. Specifically, functional connectivity within the Frontoparietal Network (FPN), the Default Mode Network (DMN), and subcortical structures involving the hippocampus were significantly able to predict these RAVLT changes. In addition, Sinha et al. [40] found a positive trend towards a significant correlation between a decline in phonemic fluency and the right pallidum and the left inferior frontal operculum connectivity after ECT. Likewise, Wei et al. [41] also evaluated cognitive function using the verbal fluency test (VFT), finding no significant difference between pre-ECT VFT scores and healthy controls scores. However, verbal fluency was significantly lower in post-ECT scores in comparison with healthy controls. However, patients with MDD showed significant correlations between the strength of the left pulvinar-bilateral precuneus connectivity and VFT scores. Moreover, Wei et al. [42] reported a significantly lower VFT score in patients with MDD after ECT compared to pre-ECT and healthy controls. Correlation analysis identified a significant positive correlation between the increased left subgenual ACC—left cerebellar lobule VI functional connectivity and the change in VFT scores in MDD following ECT.

## 4. Discussion

Given the amount of evidence supporting that the neurobiological changes following ECT may underpin its superior antidepressant efficacy as well as its associated cognitive impoverishment, we systematically reviewed all human research assessing plausible processes underlying said cognitive outcomes. It must be highlighted that although these adverse cognitive effects have been typically related to neuronal damage (such as vascular lesions or white matter hyperintensities) in worldwide clinical settings, relevant neuroimaging and biochemical studies have not found any evidence of macrostructural brain damage associated with electrically controlled induced seizures. Instead, changes at a cellular, structural, and functional level may simultaneously play a role in both the mechanism of action and adverse effects subsidiary to ECT.

The hypothesis suggesting that ECT may induce subtle neuronal or glial damage at a molecular level is supported by findings proving that the S-100 and the NSE proteins have been proved to be extremely useful to identify diffuse and small microstructural brain changes. On the one hand, the S-100 is an acidic calcium-binding protein that is present in the cytosol of astroglial cells and stimulates glial cell proliferation [43]. On the other hand, the NSE is a dimeric glycolytic enzyme that is found in the cytosol of various neuronal cells in the central nervous system (CNS) and certain neuroendocrine carcinomas [44]. Both proteins appear to be more detectable after acute CNS damage (such as ischemia, head trauma, and hemorrhagic brain injury) [43,44] and have been postulated as potential biomarkers of cognitive impairment in patients undergoing ECT. However, current research has not consistently identified S-100 protein or NSE concentrations changes significantly associated with cognitive performance after ECT. Importantly, these findings suggest that ECT-related cognitive side effects cannot be explained by an alteration of cellular structures.

Notwithstanding, other neurotoxic molecular pathways such as the Aβ amino acid peptides (involving both Aβ40 and Aβ42 subtypes) have been also proposed as potential biomarkers of ECT-induced cognitive side effects. Specifically, the Aβ42 peptide is the main component of the senile plaques in Alzheimer’s Disease (AD), and the Aβ40 peptide constitutes one of the components of the amyloid-associated brain angiopathy [45]. In this sense, reduced plasma Aβ42, increased Aβ40, and Aβ40/Aβ42 ratio changes have been reported in patients suffering from AD and MCI [46]. In addition, several preclinical studies have shown that a single injection of Aβ peptides may reduce BDNF expression in the prefrontal cortex [47]. Altogether, these findings suggest that the putative neurotoxic effects associated with plasma Aβ peptides alterations could be interfering with the BDNF-mediated neurorestorative action induced by ECT [20]. However, current literature also failed to demonstrate a robust relationship between ECT-induced plasma Aβ concentration changes and cognitive decline after ECT. Interestingly though, two studies were able to identify higher plasma S100b and Aβ40 levels at baseline as predictive biomarkers of impaired cognitive performance after ECT. Moreover, a third genetic study showed that certain BDFN polymorphisms may predict a lower risk of ECT-related anterograde memory impairment. Therefore, future research should further evaluate this preliminary predictive capability of plasma S100b protein, Aβ40 peptide, and BDFN polymorphisms to identify cognitive vulnerability profiles in patients with MDD treated with ECT. Importantly, these findings would be still in agreement with the idea that modern ECT (with seizure control, muscle relaxation, hyperoxygenation, and ventilation whenever appropriate) does not result in any detectable damage to brain tissue.

Furthermore, neuroimaging research has emerged as one of the most promising areas to identify interindividual neurobiological differences among several neuropsychiatric populations. Indeed, our current understanding of the neurobiological basis of ECT has been mainly based on advances in brain imaging studies. In this sense, previous structural neuroimaging studies have specifically highlighted the relevant involvement of hippocampal volume changes in the mechanism of action of ECT [33,48,49,50]. Interestingly, the hippocampus appears to be crucial for the antidepressant response attributable to different therapeutic modalities [51], but it is also intrinsically linked to cognitive processes such as memory and executive functions [52]. Indeed, current research has reported that hippocampal volume increases in the initial phases of ECT treatment may lead to significant cognitive impairment, while hippocampal volume decreases after 6–12 months of follow-up were associated with cognitive recovery. Although these structural neuroimaging studies do not allow for a deeply molecular understanding of the nature of these hippocampal volume increases, Cano et al. 2017 [48] suggested that both neuroplastic and neuroinflammatory changes may account for the ECT-induced clinical improvement and cognitive side effects. Likewise, several functional brain imaging studies support that ECT-induced neural functional connectivity changes between a variety of brain networks are directly related to its high efficacy in alleviating depressive symptoms [13] but may simultaneously induce a significant impoverishment of cognitive performance [2]. Importantly, functional connectivity measures within/between the salience, the frontoparietal, and the default mode networks as well as between core subcortical structures such as the hippocampus could be used as a starting point to identify connections potentially able to monitor verbal memory performance after a course of ECT.

Summarizing, despite the growing knowledge regarding the potential mechanisms underpinning the cognitive drawbacks of ECT, there are still plenty of areas that remain uncertain. Knowing that the historical use of induced seizures for the management of psychiatric disorders was initially based on the observation of a higher number of glial cells in epileptic patients gives strength to the role of seizure in preserving glial density [53]. Considering the fact that inducing generalized seizures similar to the ones occurring in epileptic patients is essential to obtain a clinical response following ECT, recent studies propose other considerations that could also play a role in the effectiveness of said controlled convulsions, such as different stimulus intensity or electrode placement, thus implying potentially improved clinical responses. Although the cognitive impairments underlying spontaneous seizures in epileptic patients have widely been explored, an interesting field to be explored could be the neurobiological similarities observed in both conditions, as well as an improvement and optimization of variables regarding the implementation of the ECT.

Finally, we would also like to highlight a promising field that is raising interest, namely the potential link between inflammatory parameters and its impact on clinical response [54]. Nevertheless, a plausible hypothesis could relate those inflammation states to the ECT-derived cognitive side effects and could also shed a light on new interesting fields of research.

## 5. Conclusions

Since ECT is a well-known effective treatment option for patients with MDD, cognition disparities subsequent to the technique still mean a significant disadvantage. Even though intrinsic characteristics of ECT implementation or different medication status could clearly play a role in the neurobiology of ECT-related cognitive disturbances, our systematic review collected all the evidence needed to develop an initial neurobiological framework for future research in the field of ECT as an effective treatment for patients with MDD, especially among those patients in whom conventional strategies have failed to succeed. We not only emphasized that ECT does not induce neuronal damage but also pointed out biochemical, genetic, and neuroimaging measures as potential predictive biomarkers of ECT-induced cognitive impact.

In the era of personalized medicine and international consortia, such as The Global ECT-MRI Research Collaboration (GEMRIC) [55], the Genetics of Electroconvulsive Therapy International Consortium (Gen-ECT-ic) [56], and the Clinical Alliance and Research in ECT Network (CARE) [57], we have an exceptional opportunity to further evaluate the neurobiological underpinnings of ECT-related cognitive side effects.

## Figures and Tables

**Table 1:** Genetic and molecular biomarkers studies.

Study	Measure	N	Diagnosis Gender/Age (Mean, Years) Medication Status	ECT Parameters	Time-Points	Cognitive Scales	Significant Results	Quality Index
Agelink et al. 2001	NSE, S-100	14	MDD, Schizoaffective depression 5W, 9M/54 Y NM (only lorazepam)	Not described	1 day before ECT, 6 h, 24 h, and 48 h after the 1st to 3rd ECT and 24 h after the 4th, 5th, 6th, and last ECT	IMC, a subtest of Blessed dementia scales (orientation, memory, and concentration), digit span test, and A-E SKT	Patients with higher post-ECT S-100 values showed better cognitive performance	4
Palmio et al. 2009	NSE, S-100b	10	Unipolar depression (with psychotic symptoms) 7W, 3M/56 Y SM	BFT, brief 3.8 sessions	Pre-ECT and 1 h, 2 h, 6 h, 24 h, and 48 h after	MMSE	-	5
Kranaster et al. 2014	NSE, S-100	19	Unipolar and bipolar depression 11W, 8M/66 Y	9.4 sessions	Pre-ECT, 30 and 60 min after the 3rd ECT and post-ECT	MMSE	Pre-ECT NSE concentration was negatively correlated with MMSE scores at baseline	5.5
Arts et al. 2006	S100-beta	12	Unipolar and bipolar depression 8W, 2M/54 Y SM	BFT, brief 6 sessions 2/week	Before ECT, 1 h and 3 h after ECT	MMSE, 15-Word Learning Task, Memory Comparisons Task, Concept Shifting Test, Letter-Digit Modalities Test, Stroop Color-Word Test, Fluency Task, CFQ, and SCL-90	S100-b levels increased 1 h and 3 h after ECT; higher S-100b concentration at baseline was associated with poorer memory function at 5 and 30 days of follow-up	6
Piccinni et al. 2013	Aβ40, Aβ42	25	Bipolar depression 12M, 13W/44 Y SM, STOP ST	BFT, brief 8.3 sessions 2/week	Pre-TEC, 1 week after last ECT	MMSE	Aβ40 levels negatively correlated with pre- and post-ECT MMSE scores; Aβ40/Aβ42 negatively correlated with post-ECT MMSE score	6
Kranaster et al. 2016	Aβ42	12	Unipolar and bipolar depression 7W, 5M/59 Y SM	RUL 10.6 sessions 2–3/week	Pre-ECT and 1–7 days post-ECT	MMSE	-	6
Yamazaki et al. 2017	Aβ40, Aβ42	42 (13 ECT)	Unipolar and bipolar depression 31W, 11M (9W, 4M ECT)/69 Y SM	BFT, brief 3/week	Pre-ECT, 2–4 weeks after ECT	MMSE, CDR, Logical Memory I and II subscales of WMS-R, Wisconsin Card Sorting Test and VFT	Aβ40 levels before discharge were negatively correlated with VFT scores; Aβ40 levels on admission were significantly higher in MCI-non-reversors compared with cognitively preserved patients or MCI-reversors	7
Bousman et al. 2015	COMT, DRD2, BDNF, APOE	117	Unipolar and bipolar depression 42% M/48 Y	BFT, BF, RUL, PW 0.3–1 ms 9 sessions 3/week	1–3 days after ECT	MCG, HVLT-R, VFT, Cross Out task, SDMT, AMI-SF, and WTAR	Interaction between DRD2 C957T and BDNF Val66Met polymorphisms on anterograde memory	5.5
Ryan et al. 2019	TL	180 (100 P)	Unipolar and bipolar depression P (62W, 38M), C (54W, 26M)/54 Y SM	BFT, RUL 8 sessions	Pre- and post-ECT	Time to recovery orientation, MMSE and CAMI-SF	-	7
Neylan et al. 2001	Cortisol	16	Unipolar and bipolar depression 11W, 5M/49 Y SM	RUL, brief pulse 6 sessions 3/week	1 day before ECT (saliva—8 A.M., 4 P.M., and 10 P.M.) and cognitive post-ECT assessment 1 day after ECT	Mattis Dementia Rating Scale, TMT, Stroop Color and Word Test, SDMT WMS subtest of Visual Recall, Employee Aptitude Survey and CVLT	Higher cortisol levels predicted ECT-induced cognitive dysfunction	5

AMI-SF, Autobiographical Memory Interview—Short Form; BFT, bilateral fronto-temporal; CAMI-SF, Columbia Autobiographical Memory Interview—Short Form; CDR, Clinical Dementia Rating; CFQ, Cognitive Failure Questionnaire; CVLT, California Verbal Learning Tes; HVLT-R, Hopkins Verbal Learning Test—Revised; IMC, Information memory concentration test; M, man; MCG, Medical College of Georgia Complex Figure Test; MMSE, Mini-Mental State Examination; NM, no-medication; NSE, Neuron-specific enolase; RUL, right unilateral; SCL-90, Symptoms Checklist 90; SKT, Syndrom Kurztest; SM, Same medication; SDMT, Symbol Digit Modalities Test; MDD, Major depressive disorder; ST, Mood-Stabilizers; VFT, Verbal Fluency Test; TMT; Trail Making Test; W, woman; WMS-R, Wechsler Memory Scale-Revised; WTAR, Wechsler Test of Adult Reading; Y, Years.

**Table 2:** Structural magnetic resonance imaging studies.

Study	Measure	N	Diagnosis Gender/Age (Mean, Years) Medication Status	ECT Parameters	Time-Points	Cognitive Scales	Significant Results	Quality Index
Figiel et al. 1990	WMH	36	MDD (ECT-induced delirium)	BFT, brief 3/week	Post-ECT	Mental Status Examination by Strub and Black	WMH within the basal ganglia after ECT	4.5
Oudega et al. 2015	WMH, MTLA, GCA	39	MDD 26% W/73 Y NM	RUL (18 switch BFT) 2/week	Pre-ECT	IQ CODE	-	4.5
Wagenmakers et al. 2021	WMH, MTLA, GCA	80	LLD 54% W/73 Y NM	RUL, BFT 11 sessions 2/week	Pre-ECT	MMSE	Worse cognitive functioning before ECT in patients with severe WMH	5
Diehl et al. 1993	MTL and thalamic T2 relaxation time	6	MDD 1W, 5M/21–55 Y Without APS	RUL, brief 3/week	1–2 days before ECT, 1 day before 2nd ECT, and 2–2.5 h post 2nd ECT	TMT, Temporal Orientation Test, Benton Visual Retention Test, HVLT, In-house verbal retrograde memory test	Post hoc correlational analyses revealed a significant relationship between left thalamus T2 relaxation time increases and ECT-induced verbal anterograde memory impairment	5
Kunigiri et al. 2007	Thalamus, hippocampal, MTL, and DLPFC T2 relaxation time	15	Melancholic depression 8W, 7M/32 Y NM	BFT, RUL 3/week	48 h after 1st ECT and 2 h after 2nd ECT	OBT, TMT-A, WMS, VLT and BVRT	-	5.5
Gbyl et al. 2019	Cortical thickness and hippocampal volume	18	Unipolar and bipolar depression 10W, 8M/47 Y SM	BFT, brief 11.9 sessions 3/week	2 days pre-ECT, 2 days, 6 days, and 6 months post-ECT	SCIP-D	-	6
Xu et al. 2019	Cortical thickness, surface area, and local gyrification index	23	MDD 12W, 11M/39 Y	BFT, brief 7.3 sessions 3/week	12–24 h pre-ECT and 24–72 h after ECT	AVLT	Cortical thickness increases in the left inferior parietal gyrus were positively correlated with AVLT score after ECT	6
Lekwauwa et al. 2006	Hippocampal volume	15	Unipolar depression 12W, 3M/74 Y	BFT, RUL 11.4 sessions	47 days after ECT	MMSE	The hippocampal volume mean of those patients with moderate or severe memory problems after ECT was significantly smaller compared to those patients without or mild memory problems	5.5
Nordanskog et al. 2014	Hippocampal volume	20	Unipolar and bipolar depression 10W, 2M/40 Y SM	RUL, BFT, brief 10.2 sessions 3/week	1-week pre-ECT, 1 week, 6 months, and 1-year post-ECT	RAVLT, RCFT, TMT-A, B, Stroop Test, VFT, Digit Symbol, Digit Span, and Block Design Test	Hippocampal volume increases one week after ECT and decreases 6 months after ECT; left hippocampal volume increase was positively correlated to TMT-A score improvement after ECT (disappeared after controlling for the number of ECT sessions)	6
Van Oostrom et al. 2018	Hippocampal volume	37 (19 P)	Unipolar depression P (37% M/50 Y), C (39% M/52 Y) NM	BFT, brief 17.7 sessions 2/week	1 week before ECT and 1 week after	TMT-A, B, VFT, RAVLT, WMS II, Visual Reproduction I-I, I and National Adult Reading Test	Hippocampal volume increases were correlated with ECT-induced decrease cognitive functioning	7
Gbyl et al. 2020	Hippocampal subfields volume	22	Unipolar and bipolar depression 11W, 11M/45 Y SM	BFT, brief 12.5 sessions 3/week	Pre-ECT, 1 week and 6 months after ECT	SCIP	Hippocampal subfields volume increases were associated with decline in cognitive performance during ECT while hippocampal subfields volume decreases correlated with cognitive recovery at 6 months follow-up	6

PCC, Posterior cingulate; PeriCAL, Pericalcarine cortex; pOPER, Pars opercularis; pORB, Pars orbitalis; PostCG, Postcentral gyrus; PreCG, Precentral gyrus; PreCUN, Precuneus; pTRI, Pars triangularis; RAC, Rostral anterior cingulate; rMFG, Rostral middle frontal gyrus; RAVLT, Rey Auditory Verbal Learning Test; RCFT, Rey Complex Figure Test; ROI, Region of interest; RUL, Right unilateral; SCIP, Screen for Cognitive impairment in Psychiatry; SFG, Superior frontal gyrus; SM, Same medication; SMG, Supramarginal gyrus; SPG, Superior parietal gyrus; STG, Superior temporal gyrus; TMT, Trail making test; TP, Temporal pole; VFT, Verbal fluency test; VLT, Verbal learning test; W, Woman; WMH, White Matter Hiperyintesities; WMS, Wechsler Memory Scale; Y, Years. AVLT, Auditory Verbal Learning test; BFT, bifronto.temporal; BSTS, Banks of the Superior Temporal Sulcus; BVRT, Benton Visual Retention Test; C, controls; CA, Cornu Amonis; CAR, caudal anterior cingulate; cMFG, caudal middle frontal gyrus; CODE, Cognitive Decline in the Elderly; CUN, Cuneus; DLPFC, Dorsolateral prefrontal cortex; EC, Entorhinal Cortex; FG, Fusiform Gyrus; FP, Frontal pole; GCA, global cortical atrophy; HATA, Hippocampus–amygdala-transition-area; HVLT, Hopkins Verbal Learning Test; IC, Isthmus cingulate; IPG, Inferior parietal gyrus; ITG, Inferior temporal gyrus; LFGo, Lateral orbitofrontal gyrus; LG, Lingual gyrus; LLD, Late-life depression; LOG, Lateral occipital gyrus; M, Man; MFGor, MDD, Major depressive disorder; Medial orbitofrontal gyrus; MTLA, medial temporal lobe atrophy; MTG, Middle temporal gyrus; MTL, Medial Temporal Lobe; NM; no.medication; P, Patients; ParaCG, Paracentral gyrus; ParaHIPP, Parahippocampal gyrus; PCC, Posterior cingulate; PeriCAL, Pericalcarine cortex; pOPER, Pars opercularis; pORB, Pars orbitalis; PostCG, Postcentral gyrus; PreCG, Precentral gyrus; PreCUN, Precuneus; pTRI, Pars triangularis; RAC, Rostral anterior cingulate; rMFG, Rostral middle frontal gyrus; RAVLT, Rey Auditory Verbal Learning Test; RCFT, Rey Complex Figure Test; ROI, Region of interest; RUL, Right unilateral; SCIP, Screen for Cognitive impairment in Psychiatry; SFG, Superdior frontal gyrus; SM, Same medication; SMG, Supramarginal gyrus; SPG, Superior parietal gyrus; STG, Superior temporal gyrus; TMT, Trail making test; TP, Temporal pole; VFT, Verbal fluency test; VLT, Verbal leraning test; WMH, White Matter Hiperyintesities; WMS, Wechsler Memory Scale.

**Table 3:** Functional magnetic resonance imaging.

Study	ROI	N	Diagnosis Gender/Age (Mean, Years) Medication Status	ECT Parameters	Time-Points	Cognitive SCALES	Significant Results	Quality Index
Abbot et al. 2014	Hippocampus	19	MDD 13W, 6M/65 Y 19 AD and 11 APS	RUL, BFT 11 sessions 3/week	Post-ECT	RBANS	Hippocampal FC normalization after ECT	5.5
Bai et al. 2018	Hippocampus	45	MDD 28W, 17M/38 Y	BFT 6–12 sessions 3/week	Pre-ECT and post-ECT	CVFT	Decreased hippocampal-angular FC was associated with cognitive impairment after ECT	6
Wang et al. 2019	Salience network	23	MDD 11W, 11M/37 Y	BFT, brief 3/week	Pre-ECT and post-ECT	AVLT	Functional and effective connectivity changes within the salience network correlated with delayed memory dysfunction	6
Wang et al. 2020	Whole-brain	24	MDD 11 W, 13M/37 Y STOP ST, BZD	BFT, brief 6.9 sessions 3/week	Pre-ECT, 1–7 days post-ECT and 1-month post-ECT	RAVLT	Functional connectivity within the FPN, the DMN, and subcortical structures were able to predict RAVLT changes after ECT	6
Sinha et al. 2019	Frontal and limbic lobes	17	MDD 10W, 7M/45 Y SM	BFT, brief 7.2 sessions	Pre-ECT and after 6th ECT	WMS	-	6
Wei et al. 2019	Whole-brain	28 P 20 HC	MDD 16 W, 12M/37 Y SM	BFT, brief 7.64 sessions 3/week	Pre-ECT and post-ECT	VFT	Left pulvinar-bilateral precuneus FC was associated with poor cognitive functioning after ECT	7
Wei et al. 2020	DMN	28 P 20 HC	MDD 16W, 12M/37 Y SM	BFT, brief 7.64 sessions 3/week	Pre-ECT and post-ECT	VFT	Increased left sgACC—left cerebellar lobule VI FC correlated with VFT scores after ECT	7

AD, Antidepressants; AG, Angular gyrus; APS, antipsychotics; AVLT, Auditory Verbal Learning test; BFT, bifronto-temporal; BZD, Benzodiazepines; CVFT, Category Verbal Fluency Test; DL, dorsolateral; DLPFC, Dorsolateral prefrontal cortex; DMN, Default mode network; FC, functional connectivity; FPN, Frontoparietal Network; HAMD, Hamilton Depression Rating Scale; HC, Healthy controls; HIPc, hippocampal cognitive subregion; HRSD, Hamilton Rating Scale for Depression; M, Man; MDD, Major depressive disorder; RBANS, Repeatable Assessment for Neuropsychological Status; RUL, right unilateral; RVALT, Rey Auditory Verbal Learning Test, sgACC, Subgenual anterior cingulate cortex; ST, Mood-Stabilizers; VFT, Verbal fluency test; W, Woman; WMS, Wechsler Memory Scale; Y, Years.

## Supplementary Materials

The following are available online at https://www.mdpi.com/2076-3425/11/10/1273/s1.
