# Supplementary material for: The Neurobiological Basis of Cognitive Side Effects of Electroconvulsive Therapy: A Systematic Review
Source: Brain Sci. 2021 Sep 26;11(10):1273. doi: 10.3390/brainsci11101273 (PMC8534116; doi:10.3390/brainsci11101273)
Supplement: Supplementary file 1 [file brainsci-11-01273-s001.zip › brainsci-1353195-SI.pdf]

Supplementary Materials: The following are available online at https://www.mdpi.com/article/10.3390/brainsci11101273/s1.

Quality Assessment Índex

To evaluate the relevance of each article reviewed for our main objectives we use a quality index for each study. A quality index was calculated as the average score of all items bellow-described and to avoid lack of transparency we also provide the score of each item in Table S1.

1. Treatment group. (1 = Only patients with major depressive disorder (MDD) are evaluated; 0.5 = Other diagnoses are included but an additional analysis only with the subgroup of patients with MDD is presented; 0 = Other diagnoses are included, and no MDD-specific analysis is provided).

2. Healthy participants as a comparison group. (1 = Yes; 0.5 = Yes but with sociodemographic differences; 0 = No).

3. Sample size of patients with MDD (1= n>10; 0 = n ≤10).

4. Sex and age report. (1 = Complete; 0.5 = Incomplete; 0 = Not reported).

5. Cognitive scales used of the electroconvulsive therapy (ECT) group, including both baseline and post-ECT assessments. (1 = Complete; 0.5 = Incomplete; 0 = Not reported).

6. Description of the ECT protocol, including electrode placement, dosage method and anaesthetics used as well as number and frequency of ECT sessions. (1 = Complete; 0.5 = Incomplete; 0 = Not reported).

7. Study protocol. (1 = Longitudinal assessments before and after completion of an acute ECT course; 0.5 = Longitudinal assessments after completion of an acute ECT course; 0 = Only baseline measures.

Study	Treatment group	Healthy participants	MDD sample sized (>10)	Sex and Age reported	Cognitive Measures	ECT protocol description	Study protocol	TOTAL
Agelink et al., 2001	0	0	1	1	1	0	1	4
Palmio et al., 2009	1	0	0	1	1	1	1	5
Kranaster et al., 2014	1	0	1	1	1	0,5	1	5.5
Arts et al., 2006	1	0	1	1	1	1	1	6
Piccinni et al., 2013	1	0	1	1	1	1	1	6
Kranaster et al., 2016	1	0	1	1	1	1	1	6
Yamazaki et al., 2017	1	1	1	1	1	1	1	7
Bousman et al., 2015	1	0	1	1	1	1	0,5	5.5
Ryan et al., 2019	1	1	1	1	1	1	1	7
Neylan et al., 2001	1	0	1	1	1	1	0	5
Figiel et al., 1990	1	0	1	0	1	1	0,5	4.5
Oudega et al., 2015	1	0	1	1	0,5	1	0	4.5
Wagenmakers et al., 2021	1	0	1	1	1	1	0	5
Diehl et al., 1993	1	0	0	1	1	1	1	5
Kunigiri et al., 2007	1	0	1	1	1	1	0,5	5.5
Gbyl et al., 2019	1	0	1	1	1	1	1	6
Xu et al., 2019	1	0	1	1	1	1	1	6
Lekwauwa et al., 2006	1	0	1	1	1	1	0,5	5.5
Nordanskog et al., 2014	1	0	1	1	1	1	1	6

Van Oostrom et al., 2018	1	1	1	1	1	1	1	7
Gbyl et al., 2020	1	0	1	1	1	1	1	6
Abbot et al., 2014	1	0	1	1	1	1	0,5	5.5
Bai et al., 2018	1	0	1	1	1	1	1	6
Wang et al., 2019	1	0	1	1	1	1	1	6
Wang et al., 2020	1	0	1	1	1	1	1	6
Sinha et al., 2019	1	0	1	1	1	1	1	6
Wei et al., 2019	1	1	1	1	1	1	1	7
Wei et al., 2020	1	1	1	1	1	1	1	7

MDD, Major depressive disorder; ECT, Electroconvulsive therapy.
